# Antifungal Activity of Glucosinolate-Derived Nitriles and Their Synergistic Activity with Glucosinolate-Derived Isothiocyanates Distinguishes Various Taxa of Brassicaceae Endophytes and Soil Fungi

**DOI:** 10.3390/plants12142741

**Published:** 2023-07-24

**Authors:** Zsolt Szűcs, Tamás Plaszkó, Eszter Bódor, Hajnalka Csoma, Lajos Ács-Szabó, Attila Kiss-Szikszai, Gábor Vasas, Sándor Gonda

**Affiliations:** 1 Department of Botany, Division of Pharmacognosy, University of Debrecen, Egyetem tér 1, 4032 Debrecen, Hungary; 2 Healthcare Industry Institute, University of Debrecen, 4032 Debrecen, Hungary; 3 Doctoral School of Pharmaceutical Sciences, University of Debrecen, 4032 Debrecen, Hungary; 4 Department of Genetics and Applied Microbiology, University of Debrecen, 4032 Debrecen, Hungary; 5 Department of Organic Chemistry, University of Debrecen, Egyetem tér 1, 4032 Debrecen, Hungary

**Keywords:** endophyte fungi, soil fungi, nitrile, isothiocyanate, ITC, synergy, antifungal activity

## Abstract

The glucosinolates of Brassicaceae plants are converted into bioactive isothiocyanates and other volatiles during a challenge by pathogens and other biotic stressors. However, the role of alternative downstream products with weaker potency (e.g., nitriles) is far from being fully understood. This study tested the possible synergistic antifungal interaction between various glucosinolate-derived nitriles and 2-phenylethyl isothiocyanate (PEITC) on 45 fungal strains, including endophytes from horseradish roots (Brassicaceae) and soil fungi, using an airtight system enabling the accurate study of extremely volatile antifungal agents. The median minimal inhibitory concentrations (MICs) were 1.28, 6.10, 27.00 and 49.72 mM for 1*H*-indole-3-acetonitrile (IAN), 3-phenylpropanenitrile (PPN), 4-(methylsulfanyl)-butanenitrile (MSBN) and 3-butenenitrile (BN, = allyl cyanide), respectively. Thus, nitriles were considerably weaker antifungal agents compared to PEITC with a median MIC of 0.04 mM. For the same nitriles, the median fractional inhibitory concentration indices (FICIs) of the combinations were 0.562, 0.531, 0.562 and 0.625, respectively. Altogether, 47.7%, 56.8%, 50.0% and 27.3% of tested fungal strains showed a synergistic antifungal activity (FICI ≤ 0.5) for the nitrile–isothiocyanate combinations, respectively. Hypocreales strains showed the least sensitivity towards the GSL decomposition products and their combinations. The mean MIC values for PEITC showed 0.0679 ± 0.0358, 0.0400 ± 0.0214, 0.0319 ± 0.0087 and 0.0178 ± 0.0171 mM for Hypocreales, Eurotiales, Glomerellales and Pleosporales, respectively. In addition, nitriles, especially IAN, also showed significant differences. For the same fungi, the median FICI values fell in the ranges of 0.61–0.67, 0.52–0.61, 0.40–0.50 and 0.48–0.67, respectively, depending on the nitrile. Our results suggest that glucosinolate-derived nitriles may enhance isothiocyanate antifungal activity and that they may play an active role in shaping the plant microbiome and contribute to the filtering of microbes by plants.

## 1. Introduction

Plants of the Brassicaceae (Cruciferae) family are known to possess a potent arsenal of bioactive compounds. The most important class of specialized compounds in these plants is that of the glucosinolates (GSLs) and their downstream products. GSLs are β-D-thioglucosides of O-sulfated (Z)-thiohydroximates. As they are biosynthesized from various amino acids, there is a considerable variance in GSL side chains. More than 100 GSLs are described in the literature; most structures are supported by both NMR and MS data [1]. Based on the side chain, GSLs are usually classified as indolic, benzenic and aliphatic GSLs. The latter group also includes sulfur-containing side chains as well. Their biosynthetic apparatus is relatively well characterized; the interested reader should consult one of the recent reviews [2].

**Figure 1:**
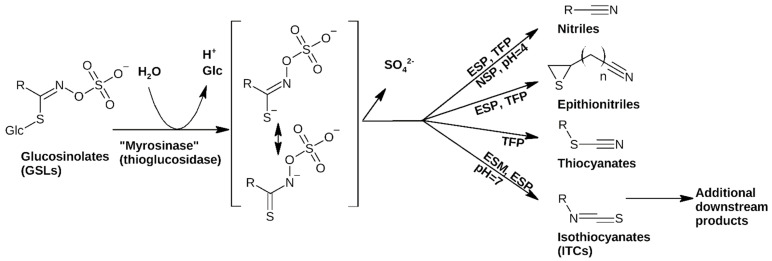
Effects of specifier proteins on the main routes of glucosinolate (GSL) decomposition. In the absence of any specifier proteins, isothiocyanates are formed. Data compiled from [5] and references therein. Abbreviation: ESP: epithiospecifier protein; TFP: thiocyanate-forming protein; ESM: epithiospecifier modifier protein; NSP: nitrile-specifier protein.

After deglucosylation by thioglucosydase (myrosinase), the resulting unstable aglycon undergoes spontaneous rearrangement to form volatile constituents; by default, these are isothiocyanates (ITCs, Figure 1), but non-enzymatic decomposition pathways have also been described [3,4]. If so-called specifier proteins are present, alternative decomposition products, such as nitriles, epithionitriles or thiocyanates, are formed (Figure 1). These primary decomposition products can be starting points for the biosynthesis of various other phytoalexins and downstream products, as we have recently discussed [5]. Furthermore, these decomposition products are thought to be the actual bioactive agents that are produced in planta, on demand. Various functions of GSL decomposition products have been covered in reviews and include activity against bacteria [6], fungi [2,7], oomycetes [6], as well as against nematodes [8] and insects [2].

The literature on nitriles and other downstream products is dwarfed by that on ITCs for most bioactivities. Plant–fungus interactions are no exception; while ITCs have been shown to have a strong antifungal effect against a wide range of fungi with various functions [7], there are many fewer reports on nitriles. The role of nitriles in fungal arrest in planta is suggested by evidence of increased biosynthesis following fungal challenge [9,10,11], and some glucosinolate-derived nitriles have also been shown to be antifungal agents in vitro against a few plant pathogen models ([5] and references therein). Nevertheless, the composition of the actual antifungal agents acting in situ remains to be fully characterized [5]; many papers have concluded that currently unknown decomposition products may also contribute to in vivo antifungal activity [12,13,14,15].

Only a few studies have been published that directly compare the antifungal activity of ITCs and other GSL hydrolysis products [5]. These have concluded that nitriles are much less potent antifungal agents when directly compared to ITCs. Not only do nitriles show lower efficacy against fungi, but insects also perform better on nitrile-producing varieties. This raises the question of why plants invest in specifier proteins to enable the production of nitriles, which appear to provide less benefit than ITCs [16].

A handful of studies provide evidence for the use of nitriles. Although 1*H*-indole-3-acetonitrile (IAN) can give rise to various downstream products such as 1*H*-indole-3-carbaldehyde and 1*H*-indole-3-carboxylic acid, these products are even weaker antifungal agents compared to nitriles [5]. In fact, they are typical fungal detoxification products of the above-mentioned compound. Nitriles could be a way for the plant to decompose GSLs without production of ITCs, thereby avoiding autotoxicity. This is relevant as downstream products of GSLs, including IAN from glucobrassicin, can also act as a biosynthetic intermediate towards generation of 1*H*-indole-3-acetic acid [17]. The ability to recover sulfur from GSLs for protein synthesis has also been recently documented, though this happens via downstream products of ITCs [18]. An additional possible role of non-isothiocyanate products could be interference with the attraction of ovipositing herbivores [16]. Other results suggest that nitriles (being volatile organic constituents) play a role in indirect defense responses; for example, the recruitment of parasitoids or predators of herbivores of Brassicaceae plants, or inter-plant communication. Exogenous application of a GSL-derived nitrile, 3-butenenitrile (=allyl cyanide), has been shown to enhance tolerance against a necrotrophic fungus, *Botrytis cinerea* in *Arabidopsis thaliana* [19]. As the downstream product mixture is sometimes dominated by nitriles [20,21,22], the above results unfortunately do not fully explain why the plant would benefit from a considerably reduced antifungal potency.

To obtain additional data on the interaction between fungi and the compounds of the glucosinolate system, we aimed to (1) assess antifungal activity of four glucosinolate-derived nitriles in a range of different fungi, (2) assess whether there is a synergistic antifungal activity between these nitriles and a model isothiocyanate and (3) assess whether any of these phenomena show differences among groups of endophytes and soil fungi, or along taxonomic groups of the strains tested. The study was carried out using endophytes from *Armoracia rusticana* (horseradish) root endophytes and soil fungi from the same site.

## 2. Results

### 2.1. Isolation and Taxonomy of Fungi Based on Their ITS Barcode

The taxonomic identification of isolates was performed using the common ITS (internal transcribed spacer) barcode [23] for the entire region, which is widely used for fungal identification. A double-level check was performed; within NCBI Genbank, we searched the RefSeq database [24] and BLAST filtered for type materials [25]. The RefSeq database contains only validated type material and associated sequences and is considered to be the most reliable taxonomically [26]. The predicted taxonomic thresholds for genus- and species-level identification of filamentous fungi based on ITS barcodes were 94.3% and 99.61%, respectively [27]. The isolated strains were uniformly identified at the genus level (Table 1). Apparently, most fungi belonged to the orders Glomerellales, Pleosporales, Hypocreales and Eurotiales.

Importantly, Glomerellales, Pleosporales and Hypocreales were the three most abundant fungal orders in our recent study on root endosphere microbiomes of field-grown horseradish (*Armoracia rusticana*), together accounting for 17.5–76.7% of all reads, while Eurotiales had a minor contribution of 0–1.6% [28]. The same orders also contributed significantly to the microbiome of Brassicales in another study comparing 33 plant orders [29].

### 2.2. Antifungal Potency of Glucosinolate-Derived Nitriles

The literature on ITCs covers fungi with various functions [7]. However, previous studies reporting on the antifungal effects of nitriles are limited to plant pathogens and most studies have only tested IAN [5]. In contrast, our approach enabled accurate determination of MIC and FICI values for compounds with very high volatility on a collection of 45 fungal strains. Airtight apparatuses are rarely used for antimicrobial studies [30,31,32,33,34], but compiled data on plant essential oil MIC values suggest that significant evaporation loss can occur in non-airtight systems [35], even with agents that are less volatile than those tested in this study. Although there is no explicit study on evaporation loss in the literature (see, e.g., a paper on yeasts [36] that found no activity for BN), our study also showed that the most volatile nitrile has the lowest potency.

While the MIC value for PEITC had a median of 0.0402 mM, the same values were 1.28, 6.11, 27.00 and 49.72 mM for the tested glucosinolate-derived nitriles, IAN, PPN, MSBN and BN, respectively (Table 1, Figure S1). This not only shows that the antifungal activity of nitriles is much lower compared to a relatively potent ITC [37], but also presents the relative potency of the indolic and aromatic nitriles compared to the aliphatic ones (MSBN, BN).

Correlation values (Spearman’s rho) between the MIC and FICI values of various strains ranged from −0.2366 to 0.6491 (Table S1). Except for PPN (which is the most similar structure to PEITC), the MIC values of nitriles showed a relatively low correlation to PEITC MIC values.

### 2.3. Synergism between ITCs and Nitriles

All four tested nitriles showed synergistic activity when combined with PEITC. The median FICI values for all fungi were 0.562, 0.531, 0.562 and 0.625 for IAN, PPN, MSBN and BN, respectively. A FICI value indicating synergy (FICI ≤ 0.5) [38,39] was found in 21, 25, 22 and 12 cases (47.7%, 56.8%, 50.0% and 27.3%) for these agents, respectively. The histogram plots of the distribution of values also show that almost all fungi showed synergistic or nearly synergistic interactions (Figure S2).

Relatively high multi-correlation (0.6034–0.6491) between the FICI values of IAN, PPN and MSBN combinations suggested similar efficacy for the nitriles of various GSL subclasses, while the BN combination was less efficient.

### 2.4. Differences among Endophytes and Soil Fungi, Fungal Taxa and Possible Microbiome Implications

**Figure 2:**
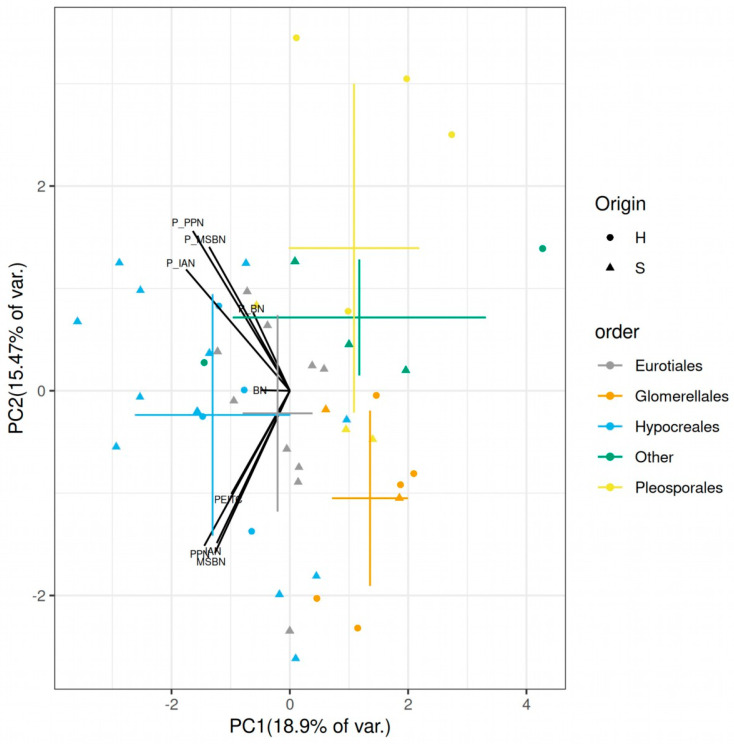
Biplot of principal component analysis scores of fungal sensitivities to glucosinolate decomposition products (nitriles and 2-phenylethyl isothiocyanate) and their combinations. Crosshairs show the average ± standard deviation for different taxonomic groups. The labeled vectors represent the relative contribution of loadings to the two principal components, PC1–2. Strains from various fungal orders are shown with different colors. Abbreviations for loading vectors: BN, IAN, MSBN, PEITC and PPN denote MIC values for individual agents; P_BN, P_IAN, P_MSBN and P_PPN denote FICI values for nitrile-PEITC combinations. Fungi of different origin are shown with circles and triangles. Abbreviations for origin: H, horseradish endophytes; S, soil.

Unlike in our study on a smaller set of fungi [37], no clear difference between the soil and endophytic fungal groups was revealed. This was mainly because both endophytes and soil fungi contained genera from various taxonomic clades, and there were groups found to be relatively distinct in their sensitivities towards various glucosinolate decomposition products. Due to the relatively multi-correlating nature of the dataset (Table S1), the separation of orders on the biplot of principal component analysis of the scaled MIC and FICI values was apparent (Figure 2); Hypocreales, Glomerellales and Pleosporales strains show distinct sensitivities (n = 16, 7, 7, respectively).

MIC values of individual GSL decomposition products varied considerably among different fungal orders (Figure S1); sensitivities to PEITC, IAN, PPN and MSBN were significantly different among fungal orders, while BN sensitivities were not (*p* = 0.0006, 0.0221, 0.0458, 0.0242 and 0.4377, respectively; Kruskal–Wallis test). Hypocreales was the least sensitive to pure compounds. Overall, the mean MIC values for Eurotiales, Glomerellales and Pleosporales were 1.03–1.69-fold, 1.11–2.13-fold and 1.05–3.81-fold lower for different compounds (Table 1). The most spectacular was the case of PEITC; Pleosporales, Glomerellales, Eurotiales and Hypocreales showed 0.0178 ± 0.0171, 0.0319 ± 0.0087, 0.0400 ± 0.0214 and 0.0679 ± 0.0358 mM for mean MIC values, respectively (*p* = 0.04658).

When checked between taxonomic orders, the FICI differences were also relatively pronounced (Figure 1 and Figure S2). The individual orders showed significantly different FICI indices for IAN, PPN and MSBN, but not for BN (*p* = 0.0073, 0.0076, 0.0102 and 0.1917, respectively, Kruskal–Wallis test). Hypocreales showed higher FICI values compared to other fungal groups. While its mean FICI values were 0.62 ± 0.16, 0.63 ± 0.16 and 0.61 ± 0.15 for IAN, PPN and MSBN, the same values for Glomerellales were only 0.44 ± 0.07, 0.40 ± 0.06 and 0.44 ± 0.05, respectively. In other words, almost all Glomerellales strains were synergistically affected by the combinations, while most of the Hypocreales strains did not show any synergy as per accepted criteria (Figure S2). The origin did not significantly affect the FICI values (*p* > 0.05), possibly because all relatively tolerant Eurotiales strains were from the soil, clustered close to the Hypocreales strains (Figure 2).

Overall, Hypocreales strains were distinguished from other clades by relatively high mean FICI values of 0.61–0.67, depending on the nitrile. In contrast, Eurotiales, Glomerellales and Pleosporales showed lower mean FICI values in the ranges of 0.52–0.61, 0.40–0.50 and 0.48–0.67, respectively.

## 3. Discussion

Although they have only been tested on a few pathogenic fungi in previous literature, the nitriles derived from indolic GSLs have been shown to be more potent antifungal agents compared to nitriles from other GSLs [5,9,10,11]. A detailed comparison of the effect of nitriles on a large number of strains is not available in the literature [5].

In the absence of specific studies, we speculate that the tested nitriles also act through aspecific target(s) and exert their toxicity via membrane disruption due to their low polarity, like other relatively nonpolar natural products of the plant kingdom [40,41]. An increase in membrane permeability might enhance the uptake of ITCs, thereby complementing their specific oxidative-stress-generating potential [7]. This is well reflected by the correlation between the logP values of nitriles and their median MIC values, which was −0.9197.

Though reports on plant volatiles acting synergistically against microbes [42,43] and a few other plant natural products [39] do exist, the direct chemical and biological interactions between the GSL decomposition products are relatively unknown and only a very limited number of available studies have tested their combinations. The homogenates of Brassicaceae species have also been shown to contain various proportions of nitriles (see Plaszkó et al. [5] and references therein). Although some of these papers also reported antifungal activity, they cannot be considered clear evidence of synergistic activity because individual compounds and their binary combinations were not tested.

Rare reports on pure compounds include descriptions of the synergistic antifungal effect of vapor phase combinations of ITCs [44,45]. Furthermore, a recent publication by Popović et al. showed synergism between PEITC, PPN and allyl ITC, involving the three fungal species *Candida albicans*, *Penicillium citrinum* and *Aspergillus niger* [46]. In the absence of binary combination tests, it is unclear whether we are seeing allyl ITC–phenylethyl ITC synergy or nitrile–ITC synergy.

Our results suggest that nitriles can be active participants in the potentiation of ITC antifungal activity under specific circumstances. Translation of the components of FICI values into real concentrations shows that synergy can occur even in mixtures with a relatively large amount of nitriles. An FICI ≤ 0.5 means that, in the presence of a relatively large amount of nitriles, a fold change of about four in ITC sensitivity can be observed. As ITCs have short half-lives due to their chemical reactivity [47,48], residual nitriles with a considerably higher stability might extend the time frame of ITC potency to some extent.

The results also explain the viability of plant accessions with nitrile-dominant GSL decomposition profiles, as the reduction in antifungal activity is likely to be much smaller than previously thought. The variance in the proportion of nitriles in the overall GSL decomposition product mixture can be extremely high, even within a species. For example, in a study on broccoli seed GSL decomposition products, the above-mentioned ratio ranged from 1.37% to 93.83% in accessions without alkenic GSLs and epithionitriles [22]. The same study found similar numbers for accessions with alkenic GSLs. In another study on *A. thaliana* accessions [21], nitriles accounted for 0–100% of the GSL decomposition products. Other studies have shown that levels of indolic GSL-derived nitriles increase after fungal challenge [9,10,11]. This supports our suggestion that nitriles or their combinations are likely to play a role in limiting fungal colonization in vivo. Although the generation of the bioactive GSL decomposition products in the vicinity of a fungal challenge site has also been documented [49,50,51], the relative proportion and in situ concentrations of various products are unknown [5].

In a more detailed analysis on *A. thaliana*, various plant accessions were challenged with *Verticillium longisporum* [20]. The authors found that the different accessions responded to the fungal challenge with different ratios of GSL decomposition product classes. Because the authors added water to lyophilized plant tissue to generate the volatiles, their results are indicative of changes in the abundance of specifier proteins [52]. While the nitrile-dominant Ler-0 showed a shift towards ITCs and a significant reduction in IAN and its 4-methoxy derivative, the isothiocyanate-dominant Hi-0 responded with increased biosynthesis of both 2-propenyl ITC and its nitrile counterpart (BN) in its leaves. The accession Bur-0 with diverse GSLs increased the production ITCs and various nitriles as well. The authors [20] speculated that nitrile levels may be more relevant in deterring fungal infection of roots. In the light of our results, a possible explanation is that *Verticillium* spp. is more sensitive to the nitrile–ITC combination than to ITCs alone. Overall, as the various accessions responded to a specific pathogen with alterations in the nitrile–isothiocyanate mixture [20], it appears that the plant is fine-tuning the synergistic mixture on demand.

The clear separation of different orders (Figure 2) shows different sensitivities to PEITC, the tested nitriles as well as their combinations. Due to the high variability in sensitivity among the 45 strains tested (Table 1, Figure S2), it is reasonable to conclude that GSL-decomposition product nitriles can modulate the plant microbiome, either alone or as adjuvants to ITCs. This leads us to the suggestion that the distinct sensitivity patterns of taxa are likely contributors to “root filtering” [53], i.e., the differences in the taxonomic composition of bulk-soil and endosphere microbiomes. The differences in the specialized metabolite patterns likely also contribute to the differences in the microbiomes of various plant families [29].

The better tolerance of Hypocreales against PEITC and the nitrile–ITC combinations also explains the findings of Ishimoto et al. [54] and those of Wand and Zhou [55]. The former group found *Fusarium* to be a dominant member of the rhizosphere of Brassicaceae plants, while the latter concluded that using *Brassica* green manures increases the relative abundance of *Fusarium* in the rhizosphere soils of cucumber. The synergistic effect might also be one of the mechanisms behind the large differences in glucosinolate–fungal-microbiome correlations in nitrile- and ITC-dominant *A. thaliana* accessions challenged by *Fusarium oxysporum* from Hypocreales [56].

Future research should take into account that the ratio and total amounts of GSL decomposition products are likely to be much better descriptors of the outcome of plant–fungus interactions than data on native glucosinolates alone. Direct testing of the effects of pure nitriles or combinations of nitriles and ITCs on soil microbiomes may provide additional insight into the relative sensitivities of fungal taxa, but to gain a deeper understanding of in vivo effects, studies comparing the microbiomes of plant accessions with different nitrile–ITC ratios are required. Even more data could be obtained from a well-designed comparison of wild-type plants with plants specifically compromised in nitrile generation.

## 4. Materials and Methods

### 4.1. Chemicals

The reagents were of analytical purity. The compounds 3-butenenitrile (BN, = Allyl cyanide), 2-phenylethyl isothiocyanate (PEITC), 3-phenylpropionitrile (PPN) and 1*H*-indole-3-acetonitrile (IAN, = indole-3-acetonitrile) were from Merck (Darmstadt, Germany), while 4-(methylsulfanyl)-butanenitrile (MSBN, = methyl thiobutyl nitrile) was purchased from ChemSpace (Riga, Latvia). The media components malt extract and peptone were obtained from Reanal (Budapest, Hungary), while glucose-monohydrate was from VWR. Dimethyl sulfoxide was from Fisher Scientific (Pittsburgh, PA, USA). Type I water was used throughout the study (18.2 MΩ), produced by a Zeneer Power I Water Purification System (Human corporation, Seoul, Republic of Korea). ACD ChemSketch v2020.2.1 was used for logP calculation.

### 4.2. Fungi

Endophytic fungi from horseradish (*Armoracia rusticana* G. Gaertn., B. Mey. & Scherb.) and soil fungi from the soil of the same site were used for this study. The isolation procedure for both endophytes and soil fungi has already been described previously [37]. Preparation of fungal inoculums was carried out as in our recent study [57]. The identification procedure was based on amplification and sequencing a characteristic barcode sequence, as previously described [37]. In addition to the fungi already identified in the aforementioned publication, a number of additional horseradish endophyte and soil fungi were identified using the ITS1-F_KYO2/ITS4_KYO3 primer pair [58]. The ITS sequences were deposited in NCBI GenBank under the accession numbers of OR019707-OR019746. All fungal strains were maintained on 2% Malt Extract Agar medium [37] during the study.

### 4.3. Antimicrobial Assay

The antifungal inhibition assay could not be conducted in 96-well plates (checkerboard assay [59]) with sufficient accuracy. In spite of testing several precautions described for studying volatile antimicrobials in [35], GC-MS evaluation of the microplate setup discovered a significant evaporation loss of several agents (BN, MSBN and, to a minor extent, PPN) within 4 days (see Supplementary Materials for additional details).

Therefore, the antifungal experiments were run in autoclaved, airtight 1.5 mL borosilicate glass screw-cap vials with 300 µL autoclaved inserts. Screw caps were disinfected using UV light. The lack of loss by evaporation was verified by a 4-day incubation study with 50 µL of diethyl ether and the agents of interest. Advantages of the current method include no evaporation loss and no cross-contamination between adjacent cells, as well as an exact in-liquid concentration of the antifungal agent.

In each vial insert, an aliquot of 196 µL Saboraud Glucose Broth (SGB, 40 g/L glucose monohydrate, peptone 10 g/L, pH 5.6 ± 0.2) was inoculated with 20 µg dry weight equivalent of fungal inoculum [37] per vial, and DMSO stocks of different agents were added so that the total amount of added volume was 4 µL. To controls, 4 µL DMSO was added. The vial sets were incubated in darkness at 20/18 °C (16/8 h). All vials were evaluated manually on day 4 for visible mycelial growth. For all fungi, at least two biological replicates were obtained for all agents, with 8–12 fungi run in parallel. Fungi with poorly reproducible MIC values for any agent, and fungi with a poorly reproducible generation of inoculation material, were eliminated from further analyses.

### 4.4. Minimum Inhibitory Concentration Measurement

For MIC determination, serial dilutions from the different agent concentrations were tested for the ability to inhibit the growth of mycelia (0.000452–0.858 mM for PEITC, 0.622–124.3 mM for BN, 0.844–84.4 mM MSBN, 0.19–48.8 mM for PPN and 0.0204–20.49 mM for IAN).

### 4.5. Synergism Experiments

The fractional inhibitory concentration index (FICI) value is considered the standard reference parameter that can quantitatively describe pairwise drug interactions in antimicrobial assays [59]. This approach was used to assess the strength of the interaction between the agents. The following conservative interpretation of FICI values [38,39] was applied: ‘synergy’ (FICI ≤ 0.5), ‘antagonism’ (FICI ≥ 4.0) and ‘no interaction’ (4.0 > FICI > 0.5).

In preliminary tests, no antagonism was found, so only combinations to assess synergies were run. In brief, after the determination of the MIC values, 0.125, 0.25 and 0.5 MIC equivalents for each agent were mixed in a 3 × 3 matrix to test growth in all combinations, so the FICI range of 0.25–1.00 was tested. As positive controls, 1 MIC concentrations from all pure agents were run in parallel. The FICI was calculated from the data of the 3 × 3 matrix using the “lowest FICI” method [60] using in-house scripts in R 4.2 [61].

### 4.6. Statistical Tests

The mean of biological replicates was submitted to all statistical tests so that data on each individual fungal-strain–sensitivity pair was considered an observation. The Kruskal–Wallis test was used to determine statistically significant differences between fungal groups for both MIC values and FICI parameters. Tests were implemented in R 4.2 [61]. When comparison of all taxonomic groups was carried out with the Kruskal–Wallis test, fungi from “other” orders were omitted from the dataset.

## 5. Conclusions

In the current work, we have shown that glucosinolate-derived nitriles act as synergistic enhancers of the antifungal efficacy of the main glucosinolate decomposition products, the isothiocyanates. This synergy was detected in approximately 50% of the tested strains, regardless of glucosinolate class. Moreover, different fungal taxa had different sensitivities towards glucosinolate-derived nitriles or their synergistic combination, as quantified by minimal inhibitory concentration (MIC) and fractional inhibitory concentration index (FICI) values. Hypocreales (many *Fusarium* strains) emerged as a more tolerant group, compared to fungi from Glomerellales, Pleosporales and Eurotiales. Thus, our study provided in vitro quantitative evidence of the effects of nitriles on various members of the plant microbiome.

This contribution of nitriles to antifungal efficacy explains the difficulty in finding actual bioactive agents that limit fungal colonization in Brassicaceae, which includes many crops and *Arabidopsis*. Although our results support the frequently described phenomenon of increased nitrile biosynthesis following fungal challenge, it draws our attention to why the plants biosynthesize different mixtures of glucosinolate downstream to cope with the challenge by different fungi.

As glucosinolate-derived nitriles and ITCs are likely to act together during fungal challenge, further studies are needed to shed light on the role of nitriles during in vivo fungal colonization of plants. Targeted studies comparing the microbiome of plant accessions with different nitrile–ITC ratios, as well as a critical comparison of the microbiomes of wild-type plants with those of plants compromised in glucosinolate-derived nitrile biosynthesis, are warranted.

## Figures and Tables

**Table 1:** Antifungal activity and synergistic potency of 2-phenylethyl isothiocyanate and its combinations with glucosinolate-derived nitriles in vitro against endophytic and soil fungi.

ID	Genus	Order	Origin	MIC (mM)					FICI (PEITC + Nitrile)			
				PEITC	IAN	PPN	MSBN	BN	IAN	PPN	MSBN	BN
F1	*Fusarium*	Hy	H	0.0536	2.56	6.11	27	79.6	0.56	0.75	0.56	0.63
F2	*Fusarium*	Hy	H	0.0804	1.28	6.11	27	44.8	0.63	0.63	0.63	1.06
F3	*Phoma*	Pl	H	0.0042	0.32	2.29	13.5	23.2	0.63	0.5	0.5	0.56
F4	*Phoma*	Pl	H	0.0067	0.43	5.09	11.3	101.9	0.63	0.75	0.75	0.75
F5	*Oidiodendron*	-	H	0.0050	0.27	1.27	15.8	49.7	0.38	0.5	0.25	0.5
F6	*Fusarium*	Hy	H	0.0536	1.28	6.11	36	29.8	0.54	0.81	0.44	0.69
F7	*Fusarium*	Hy	H	0.0804	1.28	6.11	40.5	101.9	0.56	0.5	0.5	0.52
F8	*Paraphoma*	Pl	H	0.0134	1.28	4.07	27	59.7	0.44	0.56	0.63	0.63
F9	*Plectosphaerella*	Gl	H	0.0402	1.28	4.58	27	79.6	0.44	0.38	0.38	0.5
F10	*Plectosphaerella*	Gl	H	0.0268	1.71	5.09	20.3	46.4	0.38	0.44	0.38	0.5
F11	*Pseudopyrenochaeta*	Pl	H	0.0067	0.43	2.54	13.5	41.4	0.5	0.56	0.75	0.69
F12	*Plectosphaerella*	Gl	H	0.0268	1.92	8.14	27	53	0.38	0.38	0.44	0.31
F13	*Phomopsis*	-	H	0.0201	1.28	9.16	27	79.6	0.69	0.63	0.63	0.69
F14	*Plectosphaerella*	Gl	H	0.0179	1.49	4.07	27	59.7	0.46	0.46	0.46	0.67
F15	*Plectosphaerella*	Gl	H	0.0313	1.71	7.12	36	79.6	0.5	0.31	0.46	0.54
F16	*Stagonosporopsis*	Pl	S	0.0536	0.64	6.11	27	49.7	0.44	0.44	0.5	0.5
F17	*Curvularia*	Pl	S	0.0201	1.71	6.11	27	56.4	0.38	0.56	1	0.69
F18	*Penicillium*	Eu	S	0.0804	1.28	6.11	27	79.6	0.81	0.56	0.56	0.63
F19	*Aspergillus*	Eu	S	0.0201	2.99	8.14	29.3	5	0.44	0.38	0.56	0.56
F20	*Fusarium*	Hy	S	0.0536	1.28	6.11	36	66.3	0.63	0.56	0.81	0.63
F21	*Fusarium*	Hy	S	0.1608	1.92	6.11	27	44.8	0.38	0.38	0.38	1.25
F22	*Penicillium*	Eu	S	0.0201	1.92	4.58	40.5	44.8	0.56	0.44	0.56	0.56
F23	*Fusarium*	Hy	S	0.0402	1.92	6.11	54	79.6	0.69	0.81	0.63	0.63
F24	*Penicillium*	Eu	S	0.0402	1.28	6.11	27	19.9	0.69	0.69	0.63	0.63
F25	*Penicillium*	Eu	S	0.0201	1.28	6.11	27	59.7	0.5	0.5	0.63	0.69
F26	*Fusarium*	Hy	S	0.0357	1.28	9.16	40.5	82	0.44	0.38	0.44	0.47
F27	*Penicillium*	Eu	S	0.0536	1.92	6.11	27	79.6	0.69	0.56	0.56	0.63
F28	*Penicillium*	Eu	S	0.0447	1.28	6.11	36	26.5	0.58	0.56	0.5	0.56
F29	*Fusarium*	Hy	S	0.1340	1.28	6.11	27	79.6	0.44	0.44	0.44	0.44
F30	*Penicillium*	Eu	S	0.0134	1.28	5.09	36	44.8	0.63	0.63	0.63	0.63
F31	*Cadophora*	-	S	0.0201	1.28	4.07	22.5	44.8	0.44	0.44	0.5	0.56
F32	*Clonostachys*	Hy	S	0.0402	1.92	4.58	20.3	101.9	0.63	0.69	0.69	0.57
F33	*Plectosphaerella*	Gl	S	0.0402	0.96	6.11	27	49.7	0.38	0.38	0.5	0.38
F34	*Cladosporium*	-	S	0.0201	0.64	6.11	27	43.1	0.56	0.44	0.5	0.81
F35	*Fusarium*	Hy	S	0.0804	1.92	6.11	27	79.6	0.81	0.81	0.75	0.69
F36	*Fusarium*	Hy	S	0.0804	1.92	6.11	27	59.7	0.81	0.69	0.88	0.5
F37	*Penicillium*	Eu	S	0.0536	1.28	4.58	27	29.8	0.63	0.44	0.63	0.5
F38	*Penicillium*	Eu	S	0.0536	1.92	6.11	27	19.9	0.56	0.44	0.56	0.63
F39	*Plectosphaerella*	Gl	S	0.0402	1.28	6.11	27	14.9	0.56	0.5	0.5	0.63
F40	*Cladosporium*	-	S	0.0268	0.64	6.11	27	36.5	0.63	0.5	0.63	1
F41	*Purpureocillium*	Hy	S	0.0313	1.28	6.11	27	14.9	0.5	0.44	0.5	0.75
F42	*Clonostachys*	Hy	S	0.0626	2.56	6.11	27	59.7	0.56	0.63	0.81	0.5
F43	*Aaosphaeria*	Pl	S	0.0201	1.28	6.11	27	79.6	0.38	0.56	0.56	0.38
F44	*Fusarium*	Hy	S	0.0357	2.13	8.14	36	26.5	0.69	0.75	0.69	0.75
F45	*Clonostachys*	Hy	S	0.0626	1.28	9.16	36	39.8	1	0.81	0.63	0.69

FICI values are the sum of MIC equivalents for members of the tested combination that result in inhibition of growth. Abbreviations: BN, 3-butenenitrile (allyl cyanide); IAN, 1*H*-indol-3-yl acetonitrile; MSBN, 4-(methylsulfanyl)-butanenitrile; PEITC, 2-phenylethyl isothiocyanate; PPN, phenylpropionitrile. All results are a mean of 2 measurements (nitriles’ MIC values and FICI values) or 6 measurements (MIC of PEITC). Orders: Eu, Eurotiales; Gl, Glomerellales; Hy, Hypocreales; Pl, Pleosporales; -, other. Abbreviations for origin: H, horseradish endophyte; S, soil.

## Data Availability

Additional data are available on request.

## Supplementary Materials

The following supporting information can be downloaded at: https://www.mdpi.com/article/10.3390/plants12142741/s1: Loss of volatile nitriles PPN and MSBN in parafilm-covered 96-well plates (additional description); Figure S1: Histogram showing distribution of the minimal inhibitory concentration (MIC) values of 2-phenylethyl isothiocyanate and glucosinolate-derived nitriles for fungal orders of the current study; Figure S2: Histogram showing distribution of the fractional inhibition concentration indices (FICI) values of synergistic combinations of 2-phenylethyl isothiocyanate (PEITC) and glucosinolate-derived; Table S1; Spearman’s rho correlation values between individual MIC and FICI sensitivity values; Table S2. GenBank accession numbers of fungi used throughout the study.
