# Supplementary material for: Antifungal Activity of Glucosinolate-Derived Nitriles and Their Synergistic Activity with Glucosinolate-Derived Isothiocyanates Distinguishes Various Taxa of Brassicaceae Endophytes and Soil Fungi
Source: Plants (Basel). 2023 Jul 24;12(14):2741. doi: 10.3390/plants12142741 (PMC10383044; doi:10.3390/plants12142741)
Supplement: Supplementary file 1 [file plants-12-02741-s001.zip › plants-2491250-supplementary.pdf]

Supplementary material

Loss of volatile nitriles PPN and MSBN in parafilm-covered 96-well plates.

Introduction. The initial experiments on synergy were conducted in sterile 96-well plates. While IAN - PEITC experiments were very promising, data on PPN and especially MSBN have raised doubt about retention of the entire amounts of the volatiles.

Methods. A 96-well plate with liquid media spiked with either PPN or MSBN was incubated for 4 days. Rows A-H of the plate contained 100, 200, 400, 800, 100, 200, 400 and 800 µg mL^{-1}, respectively. A multi-layer parafilm was strongly pressed onto the plate surface with a silicone 96-well plate cover mat, ensuring that the parafilm enters all cells, and the plates were incubated for 4 days under identical conditions to that described in the main paper. Thereafter, the residual nitrile amount was quantified by GC-MS.

To determine the evaporation loss, 100 µL aliquots from the plates were diluted 10 times with acetone, centrifuged, and the supernatant used for analysis directly on an Agilent 7890A GC 5975C MS instrument. Measurement parameters were as follows: split ratio was 100:1; inlet temperature was 200 °C. Time program: Initial oven temperature was 75 °C, held for 3 min, followed by a 20 °C/min temperature gradient to 240 °C and held for 4 min. An Agilent 122-0334DB-624UI 30 m × 250 µm × 1.4 µm column was used. Carrier gas was He; flow rate was 1 mL min^{-1}. Solvent delay was 9 min. As calibration curves, a serial dilution of MSBN and PPN were dissolved in acetone.

Results. In the tightly sealed 96-well plate, recoveries of MSBN from wells had a mean of 55% after 4 days, with some cells (especially 0.25 MIC cells at the edge of the plate) showing no detectable amounts (0% recovery).

In case of PPN, cells with lower concentrations absorbed PPN evaporating from other cells with higher concentrations, resulting in 2.85-fold higher concentrations than the expected amount for the 100 µg mL^{-1} well row. At the same time, 800 µg mL^{-1} cells registered an average recovery of 83% only.

Conclusions. Altogether, we have experienced significant loss of nitriles and/or cross-contamination of adjacent cells with nitriles in a tight 96-well plate system that is similar to which is shown in [54] for PPN and MSBN. Seeing these results, and bearing in mind that BN is much more volatile than the above nitriles, the 96-well plate system was rendered inadequate for our study. The phenomenon can be explained by the differences in volatility: based on data from chemspider.com, the vapor pressure of BN is 2-3 orders of magnitude higher compared to carvacrol and thymol tested in [54].

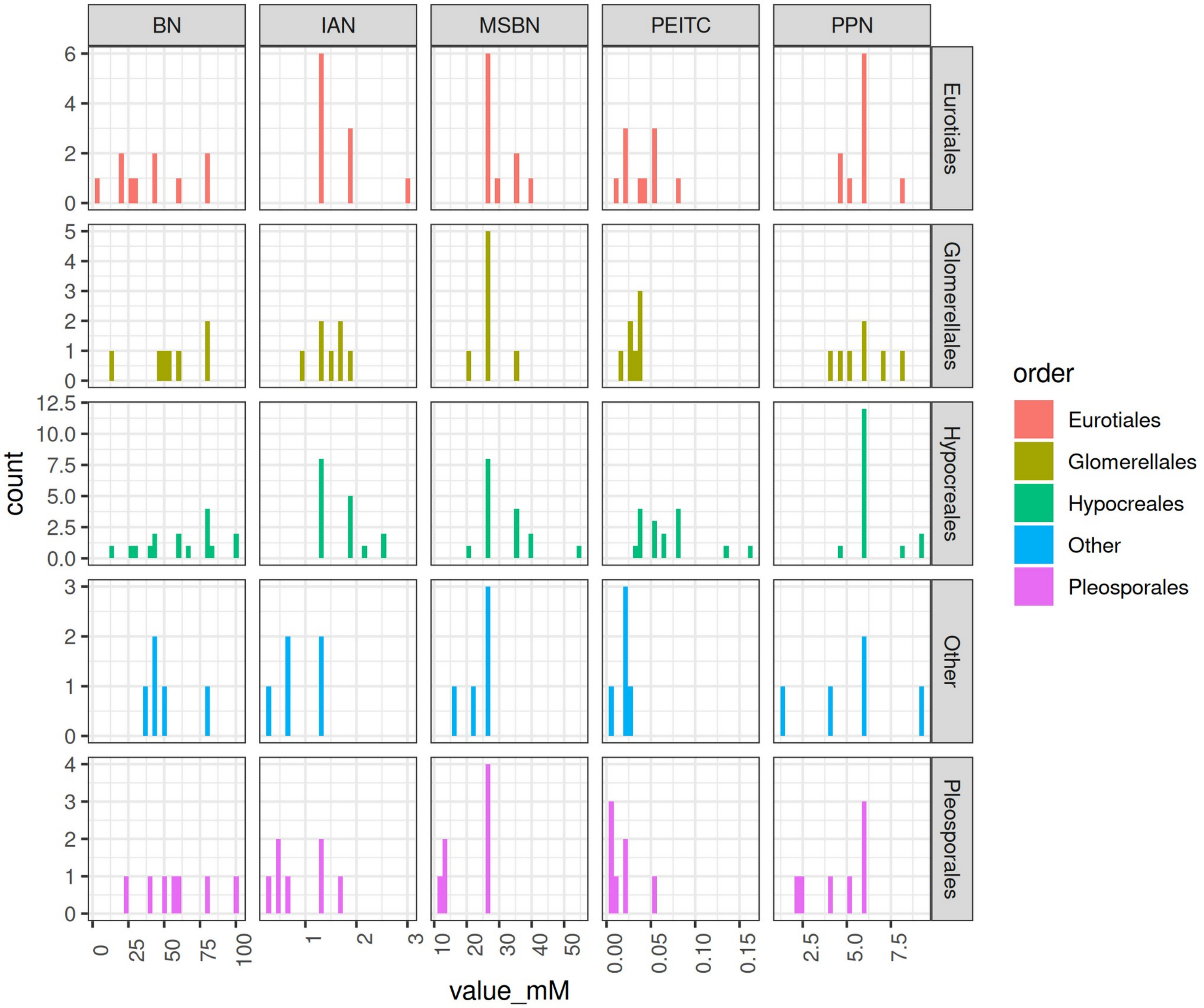

Figure S1. Histogram showing distribution of the minimal inhibitory concentration (MIC) values of 2-phenylethyl isothiocyanate and glucosinolate-derived nitriles for fungal orders of the current study. Values are given in mM. Order names are shown on the right. Abbreviations: BN, 3-butenenitrile; IAN, 1H-indol-3-yl acetonitrile; MSBN, 4-methylsulfanylbutanenitrile; PEITC, 2-phenylethyl isothiocyanate; PPN, phenylpropanenitrile.

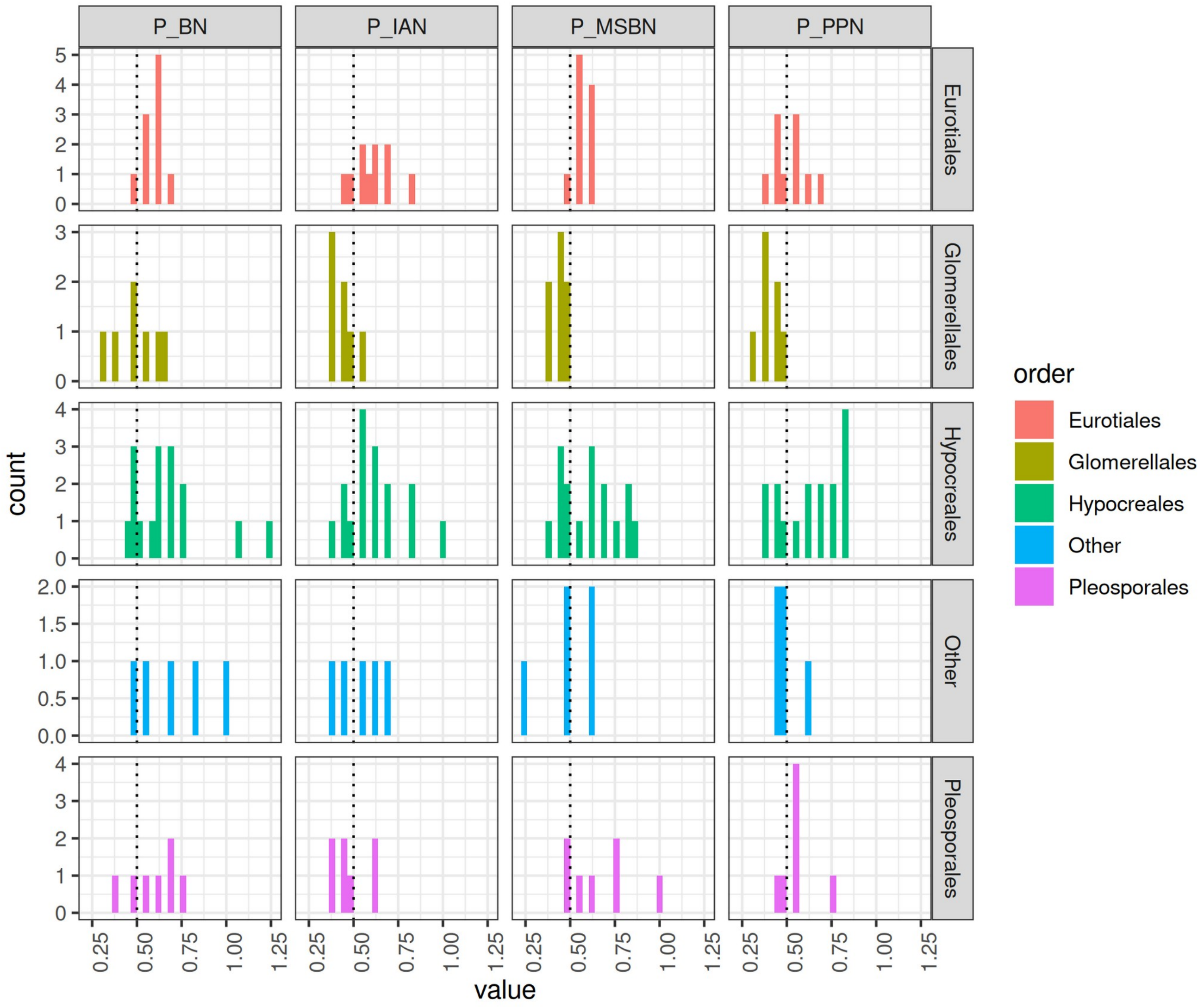

Figure S2. Histogram showing distribution of the fractional inhibition concentration indices (FICI) values of synergistic combinations of 2-phenylethyl isothiocyanate (PEITC) and glucosinolate-derived nitriles for fungal orders of the current study. Taxon names are shown on the right. Abbreviations: P_BN, PEITC + 3-butenenitrile; P_IAN, PEITC + 1H-indol-3-yl acetonitrile; P_MSBN, PEITC + 4-methylsulfanylbutanenitrile; P_PPN, PEITC + phenylpropanenitrile.

Table S1. Spearman's rho correlation values between individual MIC and FICI sensitivity values. "P+nitrile" codes refer to FICI values, other values refer for MIC. n = 45 (data for all fungi are used as averages).

	PEITC	IAN	PPN	MSBN	BN	P_IAN	P_PPN	P_MSBN	P_BN
PEITC		0.3913	0.4788	0.2930	0.2675	0.3053	0.0881	0.0249	-0.0788
IAN	0.3913		0.3599	0.2911	0.1924	0.0960	0.1182	0.1448	-0.0405
PPN	0.4788	0.3599		0.5334	0.1576	0.1733	-0.0050	0.0540	0.0246
MSBN	0.2930	0.2911	0.5334		0.0126	0.2124	0.0098	0.0357	-0.0251
BN	0.2675	0.1924	0.1576	0.0126		0.0417	0.1002	0.0584	-0.2366
P_IAN	0.3053	0.0960	0.1733	0.2124	0.0417		0.6491	0.6034	0.3522
P_PPN	0.0881	0.1182	-0.0050	0.0098	0.1002	0.6491		0.6233	0.3992
P_MSBN	0.0249	0.1448	0.0540	0.0357	0.0584	0.6034	0.6233		0.3199
P_BN	-0.0788	-0.0405	0.0246	-0.0251	-0.2366	0.3522	0.3992	0.3199	

Table S2. GenBank accession numbers of fungi used throughout the study.

ID	Genus	GenBank accession
F1	*Fusarium*	KP191628, KP191635
F2	*Fusarium*	KP191629, KP191637
F3	*Paraphoma*	KP191632, KP191639
F4	*Paraphoma*	KP191633, KP191640
F5	*Oidiodendron*	KP191634
F6	*Fusarium*	OR019707
F7	*Fusarium*	OR019708
F8	*Paraphoma*	OR019709
F9	*Plectosphaerella*	OR019710
F10	*Plectosphaerella*	OR019711
F11	*Pseudopyrenochaeta*	OR019712
F12	*Plectosphaerella*	OR019713
F13	*Phomopsis*	OR019714
F14	*Plectosphaerella*	OR019715
F15	*Plectosphaerella*	OR019716
F16	*Stagonosporopsis*	OR019732
F17	*Curvularia*	OR019733
F18	*Penicillium*	OR019734
F19	*Aspergillus*	OR019736
F20	*Fusarium*	OR019737
F21	*Fusarium*	OR019738
F22	*Penicillium*	OR019739
F23	*Fusarium*	OR019740
F24	*Penicillium*	OR019735
F25	*Penicillium*	OR019741
F26	*Fusarium*	OR019742
F27	*Penicillium*	OR019743
F28	*Penicillium*	OR019744
F29	*Fusarium*	OR019745
F30	*Penicillium*	OR019746
F31	*Cadophora*	OR019717
F32	*Clonostachys*	OR019718
F33	*Plectosphaerella*	OR019719
F34	*Cladosporium*	OR019720
F35	*Fusarium*	OR019721
F36	*Fusarium*	OR019722
F37	*Penicillium*	OR019723
F38	*Penicillium*	OR019724
F39	*Plectosphaerella*	OR019725
F40	*Cladosporium*	OR019726
F41	*Purpureocillium*	OR019727
F42	*Clonostachys*	OR019728
F43	*Aaosphaeria*	OR019729
F44	*Fusarium*	OR019730
F45	*Clonostachys*	OR019731

References

54. Houdkova, M.; Rondevaldova, J.; Doskocil, I.; Kokoska, L. Evaluation of Antibacterial Potential and Toxicity of Plant Volatile Compounds Using New Broth Microdilution Volatilization Method and Modified MTT Assay. *Fitoterapia* **2017**, *118*, 56–62, doi:10.1016/j.fitote.2017.02.008.
